# Trade and investment liberalization and Asia’s noncommunicable disease epidemic: a synthesis of data and existing literature

**DOI:** 10.1186/s12992-014-0066-8

**Published:** 2014-09-12

**Authors:** Phillip Baker, Adrian Kay, Helen Walls

**Affiliations:** Regulatory Institutions Network, College of Asia & the Pacific, Australian National University, Canberra, Australia; Crawford School of Public Policy, College of Asia & the Pacific, Australian National University, Canberra, Australia; National Centre for Epidemiology and Population Health, Research School of Population Health, Australian National University; Leverhulme Centre for Integrated Research on Agriculture and Health, and Department of Global Health and Development, Faculty of Public Health and Policy, London School of Hygiene and Tropical Medicine, London, UK

**Keywords:** Non-communicable diseases, Trade liberalization, Tobacco, Alcohol, Processed foods, Asia, Transnational corporations

## Abstract

**Background:**

Trade and investment liberalization (trade liberalization) can promote or harm health. Undoubtedly it has contributed, although unevenly, to Asia’s social and economic development over recent decades with resultant gains in life expectancy and living standards. In the absence of public health protections, however, it is also a significant upstream driver of non-communicable diseases (NCDs) including cardiovascular disease, cancer and diabetes through facilitating increased consumption of the ‘risk commodities’ tobacco, alcohol and ultra-processed foods, and by constraining access to NCD medicines. In this paper we describe the NCD burden in Asian countries, trends in risk commodity consumption and the processes by which trade liberalization has occurred in the region and contributed to these trends. We further establish pressing questions for future research on strengthening regulatory capacity to address trade liberalization impacts on risk commodity consumption and health.

**Methods:**

A semi-structured search of scholarly databases, institutional websites and internet sources for academic and grey literature. Data for descriptive statistics were sourced from Euromonitor International, the World Bank, the World Health Organization, and the World Trade Organization.

**Results:**

Consumption of tobacco, alcohol and ultra-processed foods was prevalent in the region and increasing in many countries. We find that trade liberalization can facilitate increased trade in goods, services and investments in ways that can promote risk commodity consumption, as well as constrain the available resources and capacities of governments to enact policies and programmes to mitigate such consumption. Intellectual property provisions of trade agreements may also constrain access to NCD medicines. Successive layers of the evolving global and regional trade regimes including structural adjustment, multilateral trade agreements, and preferential trade agreements have enabled transnational corporations that manufacture, market and distribute risk commodities to increasingly penetrate and promote consumption in Asian markets.

**Conclusions:**

Trade liberalization is a significant driver of the NCD epidemic in Asia. Increased participation in trade agreements requires countries to strengthen regulatory capacity to ensure adequate protections for public health. How best to achieve this through multilateral, regional and unilateral actions is a pressing question for ongoing research.

## Background

The rate of economic change in Asia over recent decades has been unprecedented. According to International Monetary Fund (IMF) data, for example, China’s share of Global GDP (PPP) rose from 2.2% in 1980 to 15.6% in 2013 [1]. India and China alone have doubled economic output per capita in less than 20 years, twice the rate achieved during the industrial revolution in the West [2]. In some countries social development has also been considerable; life expectancy in Vietnam, for example, increased from 65 to 75 and its Human Development Index (HDI) score rose from 0.44 to 0.62 between 1990 and 2011 [3]. An important driver of Asia’s economic and social progress has been the systematic reduction in barriers to cross-border trade and investment, from hereafter called ‘trade liberalization’. In recent decades, and especially since the Asian Financial Crisis, trade liberalization in Asia has accelerated in both pace and scope, initially through unilateral structural adjustment and the multi-lateral (General Agreement on Tariffs and Trade/World Trade Organization) system, and more recently through the proliferation of a ‘noodle bowl’ of preferential trade agreements (PTAs) at the bilateral and regional levels [4]. Indicative of this, bilateral or regional PTAs involving at least one Asian country increased from 46 in 1998 to 257 in 2013, of which 132 had been ratified [5]. Such processes have facilitated the development of the regions advanced cross-border production networks which underlie its status as a global ‘industrial dynamo’ [6,7].

Trade liberalization can work for or against health. On the one hand, it can promote the transfer of goods, services, investments and technologies that promote health directly through for example expanding access to nutritious foods and essential medicines, or indirectly by stimulating economic growth and employment. On the other, the benefits for health remain contested with demonstrated disparities in trade and investment related economic growth between countries, exacerbated income inequalities within countries, and heightened economic and food insecurity [8–12]. In the absence of public health protections, trade liberalization is also an important ‘upstream’ determinant of non-communicable diseases (NCDs) which for the purposes of this paper include cardiovascular disease (CVD), cancer, type-2 diabetes and chronic respiratory diseases (CRD) [13–16]. NCDs are, alongside declining yet still prevalent rates of infectious diseases, the leading causes of death and disability in Asia, and excluding Sub-Saharan Africa, in the world today [17,18].

In this paper we conceptualize trade liberalization as a driver of NCDs through two main pathways. First, it can facilitate the global diffusion of ‘risk commodities’ – tobacco, alcohol and ultra-processed foods – across borders. A key mechanism is transnational risk commodity corporations (TRCCs), those that manufacture, market and distribute such commodities on a global scale. Trade liberalization allows TRCCs to rapidly move investments, technologies, production capacity, raw materials and final products across borders and thereby drive risk commodity consumption transnationally [19–21]. Growth in risk commodity markets has stagnated in high-income countries, but is rapidly expanding in lower-middle income (L-MICs) and upper-middle income countries (U-MICs) as TRCCs seek new profit opportunities from the burgeoning middles class consumers of Asia [21,22]. Although trade remains important, foreign direct investment (FDI) is the most significant strategy used by TRCCs to penetrate new markets and grow transnationally [13,20,23,24]. Subsequently, FDI-inflows are positively correlated with risk commodity consumption rates and the prevalence of NCDs in L-MICs and U-MICs [21,25]. Since 1980 Asia has been the recipient of more FDI than any other developing region; nearly a quarter of the world’s total in 2011 [26,27].

Second, trade liberalization can strengthen the intellectual property rights of transnational pharmaceutical corporations and TRCCs. As it relates to pharmaceuticals, this can potentially constrain access to medicines and technologies used in the prevention or treatment of NCDs. While patent-ownership is concentrated in firms located in the US, EU and Japan (the top-10 largest pharmaceutical companies are located in the US and EU and have 46% of global market share) the greatest need for NCD-related medicines is located in low- and middle-income countries [28]. Trade related intellectual property rights also pertain to the protection of trademarks, brand names, product logos and trade secrets, with direct implications for policy mechanisms targeting NCD risk commodities including advertising restrictions, mandatory product labelling and product content disclosures [29].

Yet while the magnitude and implications of the NCD burden in Asia have been elucidated [17,30,31], little is known about the role of trade liberalization as a ‘cause of the causes’ of NCDs in the region [32]. Using market data and a synthesis of existing literature, this paper describes how these interconnected processes – that of trade liberalization, the activities of TRCCs and risk commodity consumption, and access to NCD medicines – are unfolding in Asia. It is structured as follows. First we demonstrate the trends and scale of risk commodity consumption across select countries in the region and elaborate on the social and economic costs of such consumption. Second we describe the processes by which trade and investment liberalization has occurred in the region to date and how it is most likely to occur in the future. In this section we describe the successive ‘layers’ of the global trade regime that have had a bearing on risk commodity consumption, including liberalization through structural adjustment programmes and unilateral liberalization, the multilateral system, and the growing importance of bilateral and regional PTAs including the Trans Pacific Partnership (TPP) agreement. We finish by outlining some pressing questions for a future research agenda on strengthening regulatory capacity to address risk commodity consumption and NCDs in the region.

## Methods

The countries included in this analysis, henceforth termed ‘Asia’, were the Association of Southeast Asian Nations plus three (ASEAN + 3) grouping of countries: Brunei Darussalam, Cambodia, Indonesia, Laos, Malaysia, Myanmar, the Philippines, Singapore, Thailand, and Vietnam (ASEAN), and China, Japan and South Korea (+3). These countries were chosen because of their participation in extensive trade and investment liberalization activities at the bilateral, regional and multilateral levels over the previous two decades. We also included India because of its large population size, economic and political importance, and because it is a significant source of patented generic NCD medicines for countries in the region. Countries were categorized by World Bank income status as high-income (H-IC), upper-middle income (U-MIC), lower-middle income (L-MIC) and low-income (L-IC). We conducted a semi-structured literature search of scholarly databases (MEDLINE, Scopus) and internet sources (Google Scholar, Google, Google Books) using a combination of three categories of search terms (Table 1) to identify relevant journal articles.

**Table 1:** **Search categories and terms used**

**Categories**	**Search terms**
Disease and risk factor specific	Noncommunicable disease, chronic disease, cardiovascular disease, diabetes, chronic obstructive pulmonary disease, cancer, stroke, obesity, hypertension, blood glucose, blood cholesterol, tobacco, smoking, alcohol, processed food, packaged food, snack food, medicine, drugs.
Economic and social burden specific	Economic, financial, income, loss, cost, expenditure, GDP, economic burden, social burden, poverty, microeconomic, macroeconomic, productivity, out-of-pocket health care, socio-economic, inequity, inequality.
Trade liberalization specific	Trade liberalization, market integration, foreign direct investment, investment liberalization, trade agreements, World Trade Organization, globalization, policy space, trade agreement, trans-pacific partnership, corporations, transnational corporations, transnational tobacco, transnational alcohol, transnational food and beverage, state-owned enterprises.

We also searched institutional websites to identify relevant grey literature and sourced detailed market reports for tobacco, alcohol and processed foods from Euromonitor International for all countries except Brunei Darussalam, Laos, Cambodia and Myanmar. Data for descriptive statistics were sourced from the World Bank World Development Indicators, United Nations Development Programme (UNDP) Human Development Index, World Health Organization (WHO) Global Health Data Repository, and WHO NCD Country Profiles. Tobacco, alcohol and processed food consumption data were sourced from Euromonitor Passport Global Market Information Database, 2013 edition, covering 1998-2012 with projections to 2017. Data were extracted for H-IC, U-MIC and L-MIC countries. Data for Brunei Darussalam, Laos, Cambodia and Myanmar were not available [33].

### The regional context

Economic growth in Asia is closely tied to the Asian epidemiological transition [31]. Economic development in Asia is likely to continue apace. By 2025, four of the world’s largest ten economies will be in the region and account for nearly half of global economic output. Seven countries are likely to lead this: China, India, Indonesia, Japan, South Korea, Thailand and Malaysia. Yet an emerging epidemic of NCDs in the region could potentially slow this ‘march to prosperity’ [6]. Some nations are also marching faster than others, with vast economic and social disparities between countries. Table 2 provides a comparison of economic, social and demographic indicators across these countries.

**Table 2:** **Country profiles including economic, social and population indicators**

**Country (ranked by descending GNI)**	**Economic and social indicators (2013) (1, 2)**				**Population (2013) (1)**			
	**World Bank Income Group**	**$GNI per capita (Atlas method, $US)**	**% living on < $2 day**	**Human Develop. Index 2013 (1990)**	**Urban (%)**	**Size (mill)**	**Life exp. birth 2011 (1990)**	**% 65 years or above (% aged 0-14)**
Singapore	High income	54,040	…	0.9 (0.76)	100	5.4	82 (76)	10 (16)
Japan		46,140	…	0.89 (0.84)	92	127.3	83 (79)	25 (13)
Brunei Darussalam		31,590^2009^	…	0.85 (0.79)	77	0.4	78 (74)	4 (25)
South Korea		25,920	…	0.89 (0.75)	84	50.2	81 (71)	12 (15)
Malaysia	Upper middle income	10,400	2.3^2009^	0.77 (0.64)	74	29.7	75 (70)	5 (26)
China		6,560	27.2^2009^	0.72 (0.5)	53	1357.4	75 (69)	9 (18)
Thailand		5,370	4.1^2010^	0.72 (0.57)	35	67.0	74 (72)	10 (18)
Indonesia	Lower middle income	3,580	46.1^2010^	0.68 (0.48)	52	249.9	71 (62)	5 (29)
Philippines		3,270	41.5^2009^	0.66 (0.58)	49	98.4	69 (65)	4 (34)
Vietnam		1,730	43.4^2008^	0.64 (0.44)	32	89.7	76 (65)	7 (23)
India		1,570	68.7^2010^	0.59 (0.41)	32	1252.1	66 (58)	5 (29)
Laos		1,460	66^2008^	0.57 (0.38)	36	6.8	68 (54)	4 (35)
Cambodia	Low income	950	49.5^2009^	0.58	20	15.1	71 (55)	4 (31)
Myanmar		…	…	0.52 (0.31)	34	53.3	65 (57)	5 (25)

Data sources: 1 = World Bank World Development Indicators; 2 = United Nations Development Programme Human Development Index.

According to World Bank data there is a 57-fold difference in GNI per capita (Atlas method) between Singapore and Cambodia. In India and Laos, more than two thirds of the population live on less than $2 per day, the World Bank’s measure of absolute poverty [3]. Rapid demographic transition is underway in many countries, with some of the highest rates of rural-urban migration and population ageing globally [31,34]. By 2030, 2.7 billion Asians or 55% of 4.9 billion, will be urbanized [34]. The region is also politically and culturally diverse. Political systems range from Marxist-Leninist Communism in Laos and Vietnam, to unitary authoritarian parliamentary systems in Singapore and Indonesia, to the world’s largest parliamentary democracy in India [35]. It is within this broad context of diversity that the NCD epidemic plays out in Asia.

### The rise of NCDs and risk commodity consumption patterns in Asia

In 2008, across the countries we include in this analysis, 17 million people died from NCDs accounting for 65% of total deaths [36]. Of these, 93% occurred in U-MICs and L-MICs primarily in China and India. This figure accounted for nearly half of the 36 million NCD-related deaths globally the same year [18]. Regional mortality patterns are given in Table 3. Although the proportion of total deaths attributable to NCDs declined with gross national income (GNI) per capita, the proportion of NCD deaths among populations under 60 years of age, or those in the most economically productive age bracket, increased [37]. In Cambodia, for example, 47% of all NCDs deaths were in this demographic whereas in Japan this figure was just 9%. Age-standardised deaths rates were also considerably higher in L-ICs and L-MICs. For example, the NCD-related death rates for males and females in the poorest country Myanmar was approximately twice that of Singapore the wealthiest.

**Table 3:** **Country NCD mortality and metabolic risk factor profiles**

**Countries (ranked by descending GNI per capita)**	**Total deaths from NCDs (000’s) (2008) (1)**			**NCD death rates per 100,000** ^*^ **(2008) (1)**		**Mortality by cause (% of total deaths, all ages) (1, 2) (2014)**							**Metabolic/behavioural risk factors, prevalence (%) (2008) (1)**									
						**CMPM**	**NCDs**	**NCDs in those <60 years old (2008)**	**CVD**	**Cancer**	**CRD**	**Diabetes**	Tobacco use* ^**†**^		**Over-weight** ^*‡^		**Obesity** ^*‡^		**Raised blood pressure** ^*§^		**Raised blood glucose** ^*¶^	
	**M**	**F**	**Both sexes**	**M**	**F**								**M**	**F**	**M**	**F**	**M**	**F**	**M**	**F**	**M**	**F**
Singapore	10.1	7.8	17.9	372	239	19	76	22	31	30	3	1	24	4	34	26	7	7	40	34	8	6
Japan	473.2	435.5	908.8	337	178	13	79	9	29	30	7	1	34	11	30	19	6	4	47	41	9	7
Brunei Darussalam	0.5	0.5	1.0	534	489	9	80	36	34	17	7	11	32	4	36	25	9	7	33	23	9	6
South Korea	112.3	96.7	209.0	465	247	8	79	19	25	30	5	4	50	5	34	29	7	8	33	28	7	6
Malaysia	50.4	39.1	89.5	606	436	16	73	31	36	15	7	3	41	2	42	46	10	17	37	32	11	10
China	4324.0	3675.4	7998.8	665	495	5	87	20	45	23	11	2	49	2	26	25	5	7	40	36	10	9
Thailand	227.1	191.3	418.4	792	541	18	71	29	29	17	9	4	36	2	27	37	5	12	36	32	7	7
Indonesia	582.3	481.7	1064.0	757	538	22	71	31	37	13	5	6	53	3	16	26	3	7	39	36	6	7
Philippines	175.7	133.9	309.5	712	483	25	67	41	33	10	5	6	35	8	25	28	5	8	35	30	6	6
India	2967.6	2273.8	5241.4	782	571	28	60	35	26	7	13	2	25	2	10	12	1	2	33	32	10	10
Vietnam	208.1	222.0	430.1	687	508	16	73	23	33	18	7	3	40	1	10	11	1	2	36	30	7	7
Laos	12.1	11.7	23.8	849	689	43	48	36	22	11	5	2	41	3	10	16	1	4	34	30	…	…
Cambodia	31.1	25.5	56.6	958	592	37	52	47	24	13	4	2	42	3	11	13	2	3	31	25	4	5
Myanmar	125.8	116.6	242.5	737	570	30	59	29	25	11	9	3	32	10	13	23	2	6	41	37	5	7
**Total**			**17011.03**																			

CMPM = Communicable, maternal, peri-natal and malnutrition related; CRD = Chronic respiratory diseases; CVD = Cardiovascular diseases; * = age-standardised; † = 15 years and older, currently smoking any tobacco product; ‡ = 20 years and older; § = systolic blood pressure ≥ 140 OR diastolic blood pressure ≥ 90 OR on medication for raised blood pressure; ¶ = 25 years and older, raised fasting blood glucose (≥7.0 mmol/L or on medication); Data sources: 1 = WHO Global Health Data Repository; 2 = WHO NCD country profiles (2014).

Differences between countries reflect variations in population-level determinants of NCDs. These include ageing populations, levels of educational attainment, the characteristics of urban environments, cultural and societal norms, and the extent to which the political and policy environments are protective [16,38,39]. Among the most significant determinants driving demand for risk commodities are rising income levels and rates of urbanization [18,30,37,40]. Yet these ‘demand-side’ factors can only partly explain global variation in consumption. They are less significant in countries with high levels of market penetration by TRCCs [14,29,41,42], implicating these commercial actors as important drivers of risk commodity consumption and NCDs in the region.

**Figure 1:**
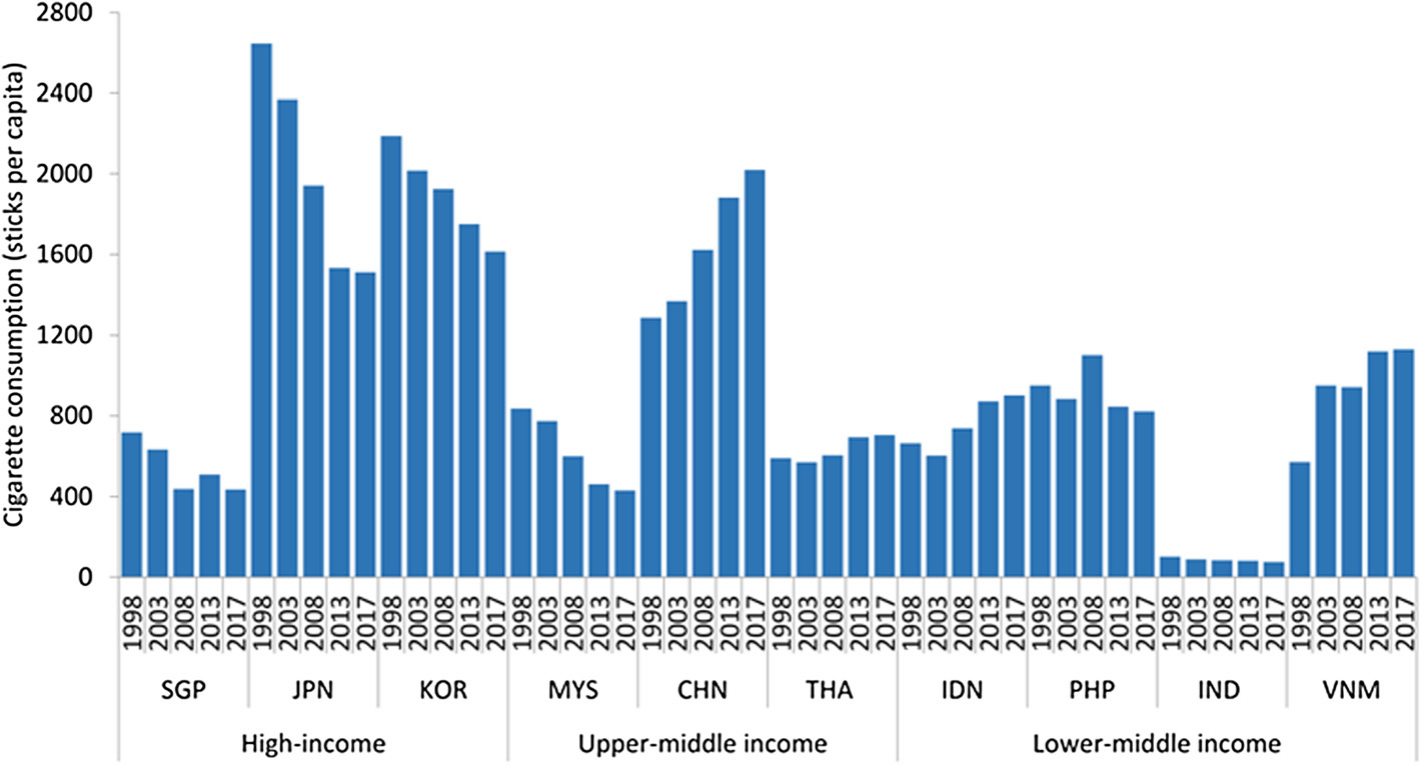
**Cigarette consumption (sticks per capita), 1998-2013 with projections to 2017, in selected Asian countries categorised by income bracket.**

Tobacco use is the most significant preventable risk factor for CVD, chronic respiratory diseases and cancers of the lung, larynx, pancreas, stomach, bladder and cervix. Smoking prevalence differs significantly from 53% among males in Indonesia to 24% in Singapore. In the H-ICs cigarette consumption is declining, but expanding rapidly in China, Indonesia and Vietnam (Figure 1). In China, consumption exceeds that of the H-ICs. It is the region’s tobacco epicentre with over 274 million daily smokers in 2012 [43]. Of these smokers 1.2 million die every year, a figure expected to rise to 3.5 million by 2030. It is also the world’s largest tobacco producer with an output of 2.5 trillion cigarettes in 2012, accounting for approximately 43% of global tobacco production [43–45]. Cigarette consumption data for India (Figure 1) should be interpreted with caution because most tobacco is consumed as bidi cigarettes which are largely manufactured in the informal sector (i.e. not captured by our market data) [46]. However, although the use of chewing tobacco is prevalent in India it demonstrates a relatively lower prevalence of tobacco use (Table 3) possibly due to its strong track record of tobacco control regulation [47,48].

All countries in the region have adopted tobacco control policies, and all except Indonesia are signatory to the Framework Convention on Tobacco Control (FCTC). In many countries including China, Thailand and Vietnam the state plays a significant role in the production, promotion and distribution of tobacco products [49]. In China for example, the market is monopolised by the state-owned enterprise China National Tobacco Corporation [50], which had a 96.9% market share in 2012 [43]. Justifications for this policy contradiction usually centre on poverty alleviation, although this view has been discredited [31,49].

These observations discount the combined role of trade liberalization and transnational tobacco corporations (TTCs) as consumption drivers in these countries. However, evidence suggests that TTCs may still promote consumption in state-dominated markets through stimulating more intensive competition (including price competition), facilitating illicit trade in foreign tobacco products and in some cases by aggressively lobbying against the adoption of public health control measures [20,51–54]. The above observations further suggest that the role of market factors as risk commodity consumption drivers is likely to be variable and context-dependent in Asia and that TTCs may promote consumption most rapidly in markets with limited state competition.

**Figure 2:**
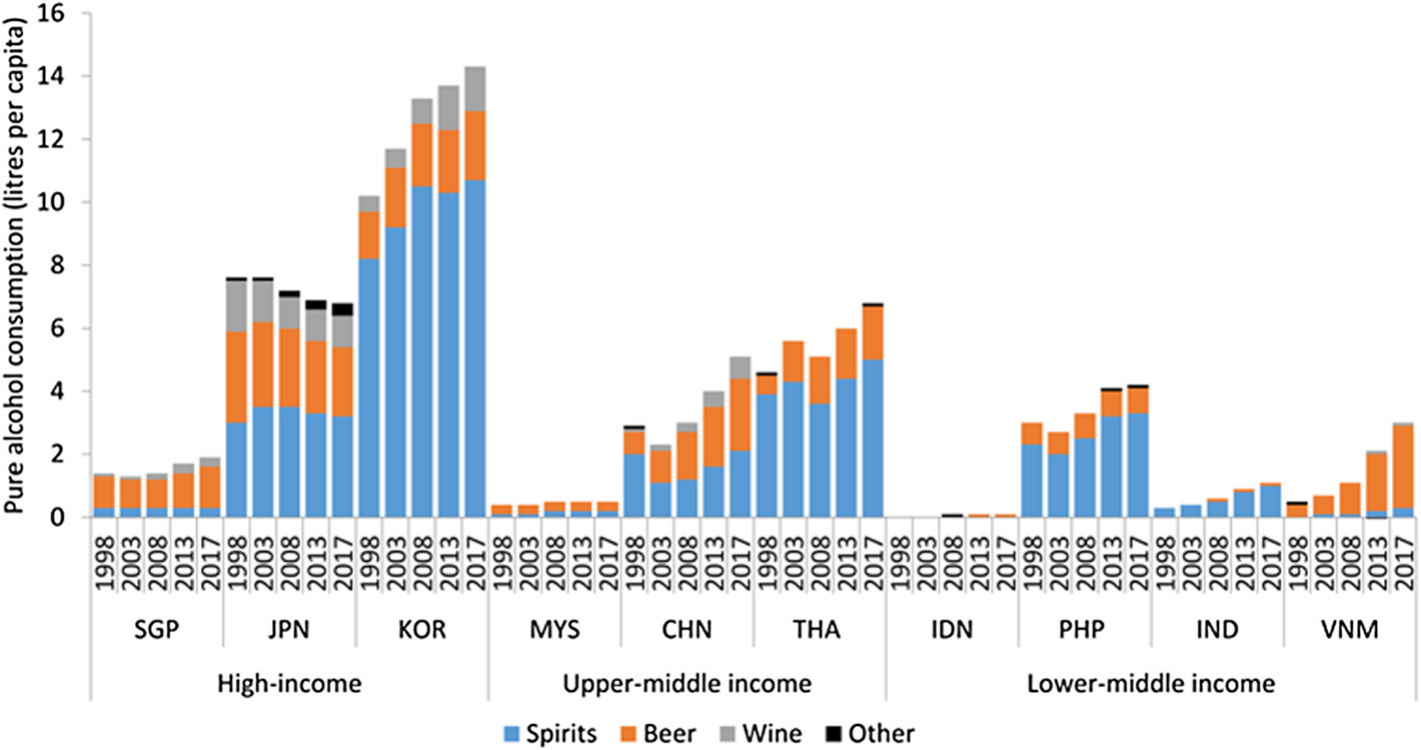
**Volume (litres per capita) of pure alcohol consumed by product category, 1998-2013 with projections to 2017, in selected Asian countries categorised by income bracket.**

The excessive consumption of alcohol is a significant preventable risk factor for CVD, some cancers, cirrhosis of the liver, road traffic injuries and some neuropsychiatric disorders [55]. Alcohol consumption is lowest in the Islamic countries Indonesia and Malaysia where there are high rates of abstention (Figure 2) [56]. Consumption in other countries is relatively low in global comparison, although the market data reported here likely underreports actual volumes as it does not capture home or craft production of traditional beverages common in many developing countries [55]. However in South Korea, China, Thailand, Philippines, India, and Vietnam our data demonstrates that alcohol consumption is increasing, primarily through consumption of spirits and beer, as well as ‘premium’ foreign brands. In China consumption of pure alcohol per capita among people 15 years or older increased 12-fold from 0.4 litres in 1952 to 4.9 litres in 2009 [57]. In the Philippines these figures were 0.7 and 4.6 litres respectively [58]. Alcohol consumption and heavy episodic drinking is higher amongst men than women in almost all countries [56]. The alcohol industry of Asia appears to be extensive. In the South-East Asia Region of the World Health Organization alone it was estimated that more than 600 factories and 1582 distributors were operating in 2003, employing more than 4 million people [59].

**Figure 3:**
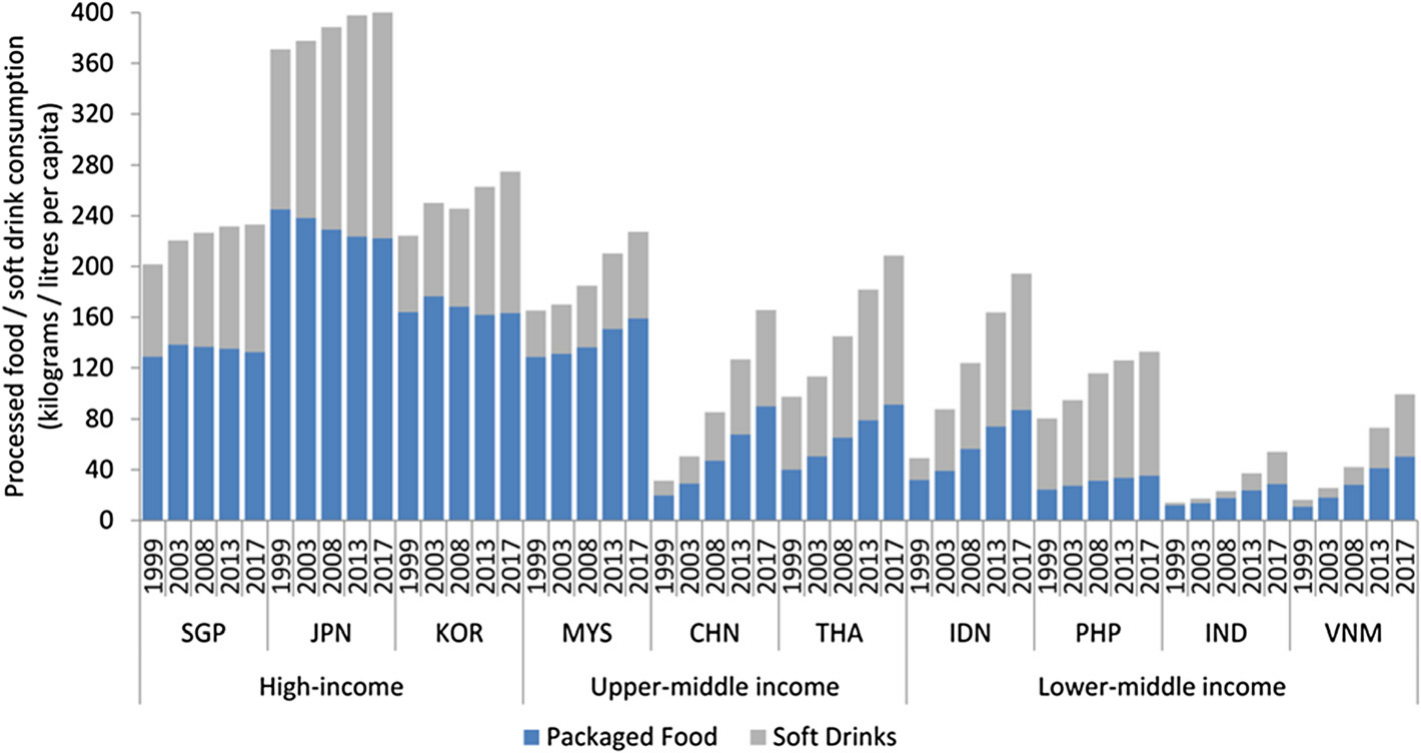
**Volume (kilograms/litres per capita) of processed foods and soft drink sales, 1998-2013 with projections to 2017, in selected Asian countries categorised by income bracket.**

Countries are also at varying stages of ‘nutrition transition’, a dietary-shift from traditional diets rich in staple foods and vegetables towards less healthy diets high in ultra-processed foods such as biscuits, confectionary, savoury snacks, processed meats and soft drinks [60–66]. As ultra-processed foods tend to be higher in sugar, salt, saturated and trans-fats relative to unprocessed or minimally processed foods, such dietary transitions are associated with rising rates of obesity and NCDs globally [13,21,65,67–70]. Although Asian countries have relatively low obesity prevalence rates, due to the high prevalence of under-nutrition in early-life stages and differences in fat patterning and cardio-metabolic effects at lower levels of adiposity, some populations may be at greater risk of diet-related NCDs in later life [65,71]. Asian populations are also more likely to develop diabetes at lower levels of obesity, at younger ages, have more complications and die at younger ages [72,73]. Although consumption of processed foods and soft drinks is highest in the H-ICs, growth rates are mostly declining or stagnating in these countries (Figure 3). In contrast, it is rapidly expanding in the U-MICs and L-MICs. In China for example, processed food consumption increased 3.2-fold from 19.6 kg per capita in 1999 to 63.4 kg in 2013. In Vietnam, it increased 3.6-fold from 10.7 kg per capita to 38.7 kg over the same period. Of concern, soft drink consumption volumes are increasing in almost all countries. Thailand, Indonesia and the Philippines appear to have volumes comparable to those of H-ICs [66].

Such trends indicate the burgeoning commercial successes of alcohol, tobacco and food industries in the context of rapidly-growing economies and rising household incomes. When the social and economic externalities of NCDs are accounted for they also constitute market failures. Extensive macroeconomic losses can result from increased health care expenditures, reductions in workforce participation, productivity and consumer spending [38,74–76]. Cumulative GDP losses related to NCDs between 2006-2015 were estimated at US$13.81 billion in China, US$16.68 billion in India and US$4.18 billion in Indonesia [38]. While the central and state governments of India generated US$5.5 billion in excise taxes from alcohol in 2003-04, they spent an estimated US$6.2 billion managing alcohol-related harms [77].

There are also significant microeconomic implications for individuals and families living below or at the poverty-line. The poor are more likely to die prematurely from NCDs because of more limited access to treatments and medicines but also because of ineffective or non-existent NCD prevention policies [78,79]. Death or disability among men of working age can be devastating for household welfare and the livelihoods of women prematurely widowed [80]. High NCD-related medical costs also have poverty implications. For example, the risk of falling into poverty was estimated to increase by 40% among Indian households in which a household member had an NCD [74].

### Trade and investment liberalization: creating favourable conditions for TRCCs

In this next step we offer some explanations as to why risk commodity consumption has increased and why it might continue to increase across the region. From the literature we identified three *processes* and five *mechanisms* that link trade liberalization to NCDs through impacts on risk commodity consumption and/or access to medicines. The three processes of trade liberalization include: structural adjustment programmes and unilateral liberalization, the multilateral system (i.e. General Agreement on Tariffs and Trade/World Trade Organization), and bilateral and regional preferential trade agreements (PTAs). These processes can also be conceptualized as successive *layers* of an evolving global trade, investment and intellectual property regime that we will elaborate on in the following sections. As it relates to risk commodity consumption each of the three processes has been more or less important at successive time points and our analysis is therefore historical.

The five mechanisms that are evident in all three processes of trade liberalization are as follows. First, trade liberalization reduces tariff and non-tariff barriers to trade and thereby facilitates imports and exports of risk commodity products or the raw materials (e.g. tobacco leaf or vegetable oil) used in their production. Second, it can reduce barriers to trade in services (e.g. manufacturing, distribution and advertising) and FDI thereby allowing TRCCs to outsource their operations to domestic corporations or to acquire them (and thus grow) through mergers and acquisitions, as well as to expand their product marketing and promotional activities^a^ [13,14,81].

Third, trade agreements can, through strengthening intellectual property rights, constrain access to medicines, technologies and medical procedures used in NCD prevention or treatment, or constrain the capacity of governments to develop legislation pertaining to risk commodity advertising, labelling and composition [28,82]. Fourth, through reducing tariff revenues and imposing significant costs associated with compliance and negotiation, trade agreements can indirectly impact health by reducing the resources available to governments used to fund policy development and implementation [83,84].

Five, through mandating processes managed by third parties (e.g. the World Trade Organization) to oversee implementation of trade agreements including dispute settlement and enforcement mechanisms, the sovereignty of nation states and their autonomy to make decisions regarding population health is diminished [85]. The above and further sub-mechanisms^b^ act to constrict domestic ‘policy space’, the freedom, scope and instruments available to governments to develop policies and regulations that can mitigate NCDs [14,86]. And because trade liberalization increasingly allows transnational corporations to make global investment decisions, it expands their capacity ‘to punish and reward countries for their policy choices by relocating investments and jobs’ [87,88 pg 13, 89]. The above mechanisms therefore serve to expand the powers of TRCC’s relative to those of nation states.

### Trade liberalization via structural adjustment

In the 1980s and 1990s trade liberalization accelerated with the emergence of a global neoliberal development agenda and the structural adjustment programmes (SAPs) imposed on many countries by the International Monetary Fund (IMF) and World Bank [90]. In return for much needed finance, such SAPs required governments to pursue reductions in their fiscal deficits (including cuts to spending on health and other social services), to open their economies to trade and FDI, to privatize state-owned enterprises (SOEs), and to eliminate support for industries serving domestic markets (import substitution) in favour of export-orientated industries [90,91]. These policies also created favourable conditions for the entry of TRCCs seeking growth opportunities in emerging markets.

In India, for example, an early-1990s IMF bail-out package required the Government to undertake extensive economic liberalization that inadvertently resulted in transformations in the domestic soft drink market sector. Following the repeal of laws that prohibited the repatriation of profits to foreign countries, PepsiCo entered the Indian market in 1990 and engaged in heavy competition with Parle, a domestic company and owner of ‘Thums Up’, a cola brand with close to 85% of the soft drinks market share [81]. After a prolonged absence the Coca-Cola Company re-entered the market in 1993 through an acquisition of Parle, establishing the company as market leader [92]. The resulting marketing and price competition between these TRCCs was fierce with significant impacts on consumption. According to Euromonitor data, between 1998 and 2012 Indian soft drink sales quadrupled from 1.2 million to 4.4. million litres with a decline in the retail selling price from US$0.6 to $0.4 per litre (in fixed 2012 dollars, constant prices) [33].

Agricultural SAPs have also likely contributed directly to a ‘nutrition transition’, in particular a regional shift in vegetable oil consumption. These have pursued the development of export-orientated cash-cropping. Between 1968 and 1993 the World Bank, for example, loaned US$618 million for palm oil production projects in Indonesia and US$383 million in Malaysia [93]. Food and Agriculture Organization (FAO) data demonstrates that Indonesian palm oil production alone increased 109-fold from 181,000 tonnes in 1968 to 19,760,000 tonnes in 2010. Indonesia’s top-two palm oil export destinations were India and China, which in 2010, received 5.3 million and 2.2 million tonnes respectively. In the same countries edible oils have contributed more than any other food source to increased energy availability [13].

### Trade liberalization via the multilateral system

The second process facilitating trade liberalization has been the multilateral trade system. This accelerated in 1995 when the General Agreement on Tariffs and Trade (GATT), the set of rules facilitating the reduction of barriers to trade since 1947, was subsumed by the World Trade Organization (WTO). The WTO serves today as the central global trade negotiating platform, that unlike the GATT system preceding it, includes a dispute settlement system and legally-binding enforcement mechanism [85]. A central tenet of WTO rules is the removal of barriers to trade including tariff barriers (financial measures for restricting trade, such as import taxes) and non-tariff barriers (other laws and regulations, such as those designed to protect public health) [85]. The GATT rules were also greatly expanded beyond trade in goods to include ‘behind-the-border’ issues such as intellectual property rights, trade in services, competition policy and investment measures. We refer the reader to comprehensive reviews of the health implications of the various WTO agreements [14,94–96].

Eight^c^ of the sixty WTO agreements are highly significant to risk commodities. The General Agreement on Trade in Services (GATS) for instance opens countries to trade and investment in services, although countries can opt-in on a sector-by-sector basis. Services liberalization can potentially enable TRCCs to contract out or acquire key services at the global level, thereby integrating and establishing control over global value chains (including research & development ➔ design ➔ production ➔ processing ➔ manufacturing ➔ distribution ➔ marketing ➔ sales) [14,97]. Such value chains can be leveraged to achieve economies of scale and drive down costs of production by sourcing services from wherever they are least expensive and regulation most favourable, processes that can ultimately lead to the increased availability, affordability and consumption of risk commodities [13]. Some services can also directly contribute to expanding risk commodity consumption. For example the ‘supermarketization’ of Asia has been facilitated by the liberalization of retail services and may contribute to the increased availability of ultra-processed foods [98].

The Agreement on Trade-Related Aspects of Intellectual Property Rights (TRIPS) expanded intellectual property protections, requiring WTO members to allow patenting of pharmaceutical products and processes (mandatory patentability). Because the TRIPs agreement grants extensive patent rights to transnational pharmaceutical firms it can prevent the import or production of generic (i.e. non-branded) drugs in developing countries, thereby potentially increasing the costs or limiting the availability of essential NCD medicines [28,99]. The TRIPS provisions also protect other forms of intellectual property including trademarks, brand names, product logos and trade secrets (information, such as product content considered confidential to producers). These provisions have direct implications for key policy mechanisms targeting NCD risk commodities, including advertising restrictions, product labelling and product content disclosure [29].

Under the Agreement on Agriculture countries are obligated to reduce agricultural tariffs, domestic agricultural supports and export subsidies [14]. This may not only result in increased imports of low-cost food commodities but also render domestic agricultural producers uncompetitive, resulting in reduced production of domestic staples [100]. Domestic producers may be further weakened when TRCCs import final products from or source their supply inputs from the heavily subsidised agricultural sectors of the US and EU [89].

The elimination of technical (non-tariff) barriers to trade was also broadened through the Agreement on Technical Barriers to Trade. This ensures countries do not adopt technical regulations (mandatory requirements for product characteristics or production methods including terminology, labelling and packaging requirements) that create unnecessary barriers to trade. It recognizes the use of technical regulation for the protection of ‘public health or safety’ as a legitimate objective when it is consistent with international technical standards. These can include, for example, standards pertaining to food, alcohol and tobacco developed by relevant international organizations such as the WHO and Codex Alimentarius [101]. However, TRCCs have at times mobilized significant opposition to the development of such standards, as demonstrated during the development of the WHO’s Global Strategy on Diet, Physical Activity and Health [102].

Under GATT articles XIV and XX the protection of human health is recognized as an interpretive principle allowing countries to adopt trade restrictive measures when it is ‘necessary to protect human, animal or plant life and health’ [14]. However, it must be demonstrated that the action is both necessary to protect health but also that no other less trade-restrictive measure is available. This provision has been interpreted very narrowly in trade disputes and successful appeals on public health grounds have been limited [14,97,100].

#### GATT-era liberalization: from bilateral sanctions to multilateral enforcement

The first stage of multilateral liberalization with a bearing on NCDs occurred under the pre-WTO GATT system. While most countries included in this analysis were signatory to the GATT prior to WTO establishment, others, concerned with the protection of domestic industries from foreign competition, proceeded with a more cautious approach to determining the depth and timing of trade liberalization [50,103]. On these grounds some did not accede to the WTO until considerably later: China in 2001, Cambodia in 2004, Vietnam in 2007 and Laos in 2013.

In other countries, however, GATT-era trade disputes were significant in the liberalization of tobacco trade. Bilateral trade sanctions (non-GATT) were used as an initial mechanism. For example the US Cigarette Export Association, a tobacco industry lobby group, solicited support from the US government to threaten trade sanctions against South Korea, Japan, Taiwan and Thailand unless they opened their domestic markets to US tobacco products [104]. In the latter three countries this was successful, but to the significant detriment of public health. Only one-year after the opening of the Japanese market smoking prevalence doubled from 16% in 1986 to 32% in 1987, with most growth among adolescent girls [105]. Similarly, in South Korea smoking prevalence increased from 18.4% to 29.8% among adolescent males and from 1.6% to 8.7% among adolescent females in a one year period [104]. This is reflected in tobacco company corporate documents demonstrating the deliberate and strategic targeting of young women [106]. Thailand, however, maintained import restrictions arguing that US cigarettes contained additives more harmful than those in domestic cigarettes. Subsequently, in 1990, the US government made a successful claim that these restrictions violated GATT rules [107]. The GATT tribunal, however, permitted Thailand to maintain existing tobacco control regulations including advertising restrictions, non-discriminatory labelling, and ingredient disclosure laws [108]. As a result Thailand has one of the most comprehensive tobacco control regimes globally and has maintained one of the lowest smoking prevalence rates across the region [109]. Nonetheless, this period constituted an important shift from bilateral trade sanctions to multilateral enforcement for opening markets to tobacco trade and consumption.

#### WTO accession: liberalization through conditionality of membership

The second stage of multilateral liberalization has resulted from accession to WTO membership. In order to join the WTO a nation must declare all domestic policies that have a bearing on the various WTO agreements and is often required to modify such policies before membership is granted. On these grounds the US challenged Vietnam’s accession to the WTO claiming that its alcohol taxation policies favoured domestic over foreign producers. China and Taiwan also faced pressure during their WTO accession negotiations to privatize their state-owned tobacco monopolies [110]. British American Tobacco (BAT) the world’s most ‘transnationalized’ transnational tobacco company (TTC) strategically lobbied EU, UK and US government officials to achieve concessions from China during its WTO accession negotiations. In 2004 the Chinese Government implemented its commitments including reduced tobacco import tariffs and eliminated retail licensing (allowing retailers to sell foreign brands). These changes were associated with increased tobacco imports and reductions in foreign cigarettes prices [50], although market penetration by TTCs remains low [43].

Some nations have made certain sectors and products exempt from their WTO accession commitments. This may have also influenced the scale of risk commodity consumption. In India, for example, multi-brand retail was excluded from its GATS commitments and has been closed to foreign investment except through minority joint ventures, resulting in very low levels of penetration by transnational supermarket retailers [66,111]. Because supermarkets can act as vectors for ultra-processed food distribution [98], this may partly explain why India has the lowest levels of consumption in the region (Figure 3). Despite lengthy nationwide protests by farmers, retailers and unions, however, a new law passed in September 2012 allowing up to 51% foreign ownership in the sector will likely change the retail landscape significantly [112].

#### WTO Dispute Settlement System: liberalization through trade disputes

The third stage of multilateral liberalization has been through the WTO’s Dispute Settlement System. WTO mediated trade disputes have opened multiple countries to trade in risk commodities. Table 4 provides a simple overview of trade dispute cases made under the GATT rules pertaining to agriculture, alcohol, tobacco and pharmaceuticals between 1996 and 2013. This demonstrates that Thailand, Indonesia, Philippines and India have used these rules to promote access for their domestic producers to foreign and regional markets. For example, in 2008, the Philippines claimed that Thailand’s customs valuation procedures and comprehensive tobacco taxation scheme constituted unfair obstacles to tobacco importation [107].

**Table 4:** **Relevant trade dispute cases between 1995 and 2013 made under GATT rules**

**Country**	**Signatory to GATT**	**Accession to WTO**	**Dispute claims made (as Complainant)**				**Dispute claims against (as Respondent)**			
			**Agriculture**	**Alcohol**	**Tobacco**	**Pharma.**	**Agriculture**	**Alcohol**	**Tobacco**	**Pharma.**
Singapore	1973	1995	---	---	---	---	---	---	---	---
Japan	1955	1995	---	---	---	---	US (2002); US (1997); EC (1997)	US (1995)^†^; EC (1996)^†^; Canada (1996)^†^	---	---
Brunei Darussalam	1993	1995	---	---	---	---	---	---	---	---
South Korea	1967	1995	---	---	---	---	US (1995); US (1995) US (1996; US (1997); US (1999*; Australia 1999)*; Canada (2009)*	EC (1997)^†^; US (1997)^†^	---	---
Malaysia	1957	1995	---	---	---	---	---	---	---	---
China	---	2001	---	---	---	---	---	---	---	---
Thailand	1982	1995	EC (1995); EC (2003)^‡^; EC (2003); Hungary (1996)	---	---	---	Egypt (2000)	---	Philippines (2008)^†^	---
Indonesia	1950	1995	---	---	US (2010)	---	US (2013)	---	---	---
Philippines	1979	1995	Australia (2002)	---	Thailand (2008)^†^	---	US (1997)	EC (2009)^†^; US (2010)^†^		
India	1948	1995	---	---	---	Argentina (2001); EC; Netherlands (2010)	US (2001); US, Australia, Canada, NZL, Switzerland (1997)	EC (2008)^†^	---	US (1997); EC (1997)
Vietnam	---	2007	---	---	---	---	---	---	---	---
Laos	---	2013	---	---	---	---	---	---	---	---
Cambodia	---	2004	---	---	---	---	---	---	---	---
Myanmar	1948	1995	---	---	---	---	---	---	---	---
		Totals	5	0	2	3	15	8	1	2

Years in brackets are when request was made for consultation; Pharma. = Pharmaceuticals; * = disputes pertaining to beef importation; † = disputes pertaining to taxation; ‡ = disputed agricultural subsidies made under the Common Agricultural Policy of the EU; Data source: [118].

What is also evident, however, is that Western nations have even more frequently used WTO rules to promote access to Asian markets for food and alcohol producers. Tobacco appears to have been under-represented in WTO trade disputes, most likely because of GATT-era liberalization and bilateral sanctions we described earlier had already resulted in market entry by TTCs. Of the 26 claims made against Asian nations, 21 were made by the United States (US) and European Community (EC) alone and of these 9 were against L-MICs and U-MICs. To the contrary only 5 claims were made by L-MICs and U-MICs against the US and EC. These differences likely reflect asymmetries in markets sizes and therefore bargaining power, but also the resources available to nations to make or defend disputes which can involve often complex and costly legal proceedings [90]. Developed countries not only make more claims, they also win more [84]. One analysis demonstrated success rates of 74% and 50% for developed and developing countries respectively [113].

Most notably, the US, Canada and the EC have made claims against Japan, South Korea, the Philippines and India to reduce taxes on imported alcohol products. For example, the national treatment rule was invoked to remove Japan’s high taxes on imported alcohol products. This began with vodka as considered ‘like’ the traditional spirit shochu but would subsequently include gin, rum, whiskey, brandy and other spirits. Subsequently domestic liquor prices dropped [110], although according to our data consumption did not increase (Figure 2) and we were unable to find further information to explain this trend. The same rule was invoked by the EU in 1999 to eliminate South Korea’s preferential taxation scheme determined as favourable to domestic alcohol producers of the national spirit soju [114].

The threat of a WTO trade dispute can also result in ‘regulatory chill’, the non-adoption of domestic policies arising not from a dispute, but from the threat of one. For example, with the aim of reducing consumption of processed snack foods Thai civil society groups advocated for a law requiring that products are labelled with a ‘Children Should Take Less’ message as well as a traffic-light labelling system to indicate the sugar, fat, sodium and energy content of products. Although considered by the Thai Food and Drug Administration this was abandoned in favour of an industry preferred Guideline Daily Amount thumbnail labelling system when several WTO members raised concerns about these policies during the Technical Barriers to Trade committee deliberations [115].

#### Trade-Related Aspects of Intellectual Property Rights agreement: implications for the region

The TRIPS agreement has a significant bearing on NCD mitigation as it can determine the availability and affordability of life-saving medicines. Under ‘TRIPS-flexibilities’ there are legal mechanisms, such as compulsory licensing, available to governments to allow the production and import of patented pharmaceuticals by domestic firms [28]. Under these rules, or in despite of them, Asian countries have promoted access to medicines in two ways.

First has been through the interpretation and implementation of TRIPS-flexibilities, in particular by Thailand, Malaysia and Indonesia. Moon and Szlezak have described the actions taken by these governments in issuing compulsory licenses for HIV/AIDS drugs since the 1990s. Building off these developments Thailand more recently extended TRIPS-flexibilities to NCD drugs. In 2007 it announced a compulsory license to a domestic firm to produce Clopidogrel, a blood-thinning drug for hypertension patented by French firm Sanofi (under the brand name Plavix). In 2008, compulsory licenses were also announced for four cancer drugs: Erlotinbi, Docetaxel, Letrozole and Imatinib (the latter was not implemented due to intervention by Novartis) [116].

The second is exemplified by India through its adoption of stand-alone national patent licensing legislation that gives significant weighting to public health protection. Most developing countries usually have small or non-existent pharmaceutical industries and must therefore import patented medicines from developed countries. This often contributes to large health-cost and balance of payments deficits [28]. India is an exception. Known as the ‘pharmacy of the developing world’ it has a highly competitive domestic pharmaceutical industry that not only produces large quantities of generic medicines at very low-cost, but also exports two-thirds of production to LMICs [99]. The legality of India’s pharmaceutical sector is, for these reasons, of particular relevance to NCD mitigation across Asia.

In 1997 the US and EU bought complaints against India alleging that the absence of a patent protection system violated the TRIPS mandatory patentability rule. India used TRIPS-flexibilities and made full use of the transition period allowed for developing countries under WTO accession rules to develop its national patenting legislation. This scheme became fully operational in 2005 [117]. Unlike the Thailand and Indonesian schemes that invoke TRIPS-flexibilities in order to issue compulsory licenses on patents already recognised by their national laws, the Indian scheme can invoke TRIPS-flexibilities but also preclude the granting of patents in the first place [116].

As a result India has been the centre of a legal battleground that has broad repercussions for access to NCD medicines. Two cases pertain to cancer medication. In a first case the company Novartis was refused a patent for the leukaemia drug Imatinib, allowing Indian generic companies to supply the drug at almost one twentieth of the price (US$124-174 versus $2478 per month). In a second, although the company Bayer was granted a patent for its renal cancer drug Sorafenib (marketed as Nexavar), the Indian Government issued a compulsory license to an Indian company that allowed supply at more than one fortieth of the branded price (US$125 versus $5500 per month) [99,117].

The future of TRIPS and tobacco labelling policy in Asia appears to be untested in this space (although as described earlier, Thailand was able to maintain tobacco control measures under earlier GATT rules). However Asian nations such as Malaysia and Thailand continue as ‘observers’ of the recent WTO challenge to Australia’s plain packaging regulation bought by a number of tobacco producing countries that could set an important precedent for tobacco control in the region [118].

### Liberalization via bilateral and regional preferential trade agreements

The third process is the more recent proliferation of bilateral and regional preferential trade agreements (PTAs). While PTAs are often referred to as free trade agreements they are best described as ‘preferential’ trade agreements because they are never truly ‘free’, but rather provide signatories with more favourable (and thus preferential) terms of trade than non-signatories. While bilateral investment treaties (BITS) have been numerically more common, particularly so during the 1990s, today regional PTAs with investment provisions are more economically significant. Thus the most significant contemporary trend in the global trade regime is best described as multilateralism with ‘regionalism on the rise’ [26].

Regional and extra-regional economic integration in Asia greatly accelerated following the 1998 Asian Financial Crisis [4]. PTAs involving at least one Asian country increased from 46 in 1998 to 257 in 2013. Of these 50 were in the proposed stage, 75 were under negotiation and 132 had been signed. One possible reason for this growth in PTAs is that they provide a mechanism for accelerated trade liberalization as an alternative to the slow-pace of multilateral (i.e. WTO) negotiations [119]. This may also constitute a commercial strategy, known as ‘forum-shifting’, that offers TRCCs and their representative governments an alternative mechanism for opening markets to trade and investment [5]. Initial agreements may also trigger a domino-effect as other countries initiate further PTAs to retain trade competitiveness [119].

The second important trend is that PTAs are becoming increasingly ‘deep’ with commitments and concessions that go beyond those required by the WTO system [120]. First they include provisions *consistent with* the existing WTO agreements but also commitments that go beyond them, thus referred to as ‘WTO-plus’ (agreements also take this nomenclature e.g. ‘TRIPS-plus’). They also include commitments *outside* of the current WTO framework, referred to as ‘WTO-X’ including those on intellectual property rights, investment and competition [120]. These are not so much concerned with facilitating ‘cross-border’ trade but with integrating ‘behind-the-border’ domestic policies that represent threats to global intra and inter-firm supply chains [121].

The implications for public health have generated significant concern for a number of reasons. First, while the multilateral system provides flexibilities on public health and safety grounds these can be excluded from or highly restricted within PTAs [14,28]. Second, while under the multilateral system trade disputes are made by one government against another, investor-state dispute settlement (ISDS) mechanisms in many PTAs allow corporate investors to file legal proceedings directly against governments to recuperate losses resulting from the adoption of domestic regulations (including health regulations) [29]. Third, with significant asymmetries in economic and therefore bargaining power, developing countries are more likely to make deep concessions in PTA negotiations; to date this has been most evident within investment and intellectual property provisions of such agreements. Finally, PTA negotiations are usually ‘closed door’, therefore lacking the greater transparency of multilateral negotiations [122].

The net effect is to not only further constrain the domestic public health policy mechanisms available to signatory governments (i.e. reduce their ‘policy space’), but to also greatly expand the power of TRCCs and representative governments to dispute policies enacted by governments to prevent or control NCDs. Restrictions on risk commodity advertising could, for example, be represented as barriers to cross-border trade in advertising services. Compliance costs and evidence requirements imposed on countries aiming to regulate advertising could also act as a deterrent. The South Korea-US PTA (KORUS), for example, contains stringent criteria on countries wishing to regulate the supply of services. This concern has been flagged as a potential threat to legislation targeting the marketing of foods and beverages to children [82,115].

The impacts of PTAs on NCD risk factor consumption can be significant. A recent analysis demonstrated that a PTA with the US was associated with a 63.4% higher soft drink consumption per capita compared to countries with no US PTA [21]. This is of concern given an association between soft drink consumption, obesity and type-2 diabetes [123]. The North American Free Trade Agreement (NAFTA) has been particularly significant in the case of Mexico. A combination of subsidised agricultural production in the US (propagated by the US Farm Bill), and increased processed food exports and FDI by US firms into Mexico have had deleterious impacts on public health nutrition [124,125]. Mexicans now consume more than 300 litres of soft drinks per capita per year, the highest consumption globally. In 2011, 32.4% of Mexican adults were reported as obese, the second highest rate among OECD countries [21].

Several PTAs are or will be notably significant to NCDs in Asia. The 2003 US-Singapore agreement eliminated tobacco tariffs and included an ISDS allowing investors to directly challenge government regulations [110]. KORUS is another. It opened South Korea to US agricultural imports, most significantly for beef, previously limited on public safety grounds following fears over bovine spongiform encephalopathy (mad-cow disease). The US trade negotiator was also pressured by a diversity of business groups, including alcohol and tobacco companies, to not exclude any service, sector or product from the agreement [29]. KORUS was an important agreement, not only because South Korea made ground-breaking concessions but also because it now serves as a template for future US-led trade negotiations including the Trans-Pacific Partnership Agreement (TPP) currently under negotiation [126].

#### The Trans-Pacific Partnership Agreement

In economic terms the TPP has the potential to be the most significant trade policy of the 21^st^ Century. The twelve negotiating countries include Australia, Brunei, Canada, Chile, Japan, Malaysia, Mexico, New Zealand, Peru, Singapore, the US and Vietnam. South Korea may later join the negotiations. Together these counties have an aggregate GDP of US$28 trillion (40% of the world total), and were attributed to 27% of world exports worth US$6 trillion in 2011. The TPP is also significant because it serves as an interim arrangement prior to a more comprehensive Free Trade Area of the Asia Pacific (FTAAP) that may incorporate a greater number of countries [127].

The TPP constitutes a significant threat to public health policies targeting risk commodities in the participating countries of Asia [115,121,126]. Although some countries have proposed that the agreement should ‘support each Party’s right to protect public health, including by facilitating timely access to affordable medicines’ and that countries can ‘adopt measures necessary to protect public health and nutrition’ the US and Japan have opposed adoption of these provisions [As quoted in 131]. A leaked draft text demonstrates that investment provisions of the TPP do not allow any general exceptions on health and consumer protection grounds [115,129].

Other key provisions of concern include those on regulatory coherence and transparency that require governments to consult industry and disclose information during domestic policy-making processes [115]. Unlike the WTO system where arbitration is between nation-states, the TPP draft text includes an ISDS mechanism that investors (corporations) can utilize to pursue compensation from governments for losses resulting from the adoption of health regulations [121]. Although these have been incorporated into PTAs for some time, Philip Morris Asia has recently pursued compensation from the Australian Government for its plain packaging tobacco legislation using an ISDS provision within Australia’s Bilateral Investment Treaty with Hong Kong [130]. The threat of similar future arbitration from TRCCs may promote regulatory chill among domestic policy-makers (i.e. reluctance to adopt a given policy mechanisms through fear of an investor dispute) and thereby constrain future innovation in public health policy targeting risk commodities [121,127]. The example also indicates that TRCCs can leverage PTAs from whichever country they have a subsidiary and from wherever their legal standing most favourable.

Intellectual property rights (IPR) provisions of the TPP are also highly contentious. The US has pushed for a broad approach to IPR akin to the KORUS agreement on patents, copyrights, trademarks and trade secrets. This includes demands that animals, plants and medical procedures are subject to patent protection [128]. US negotiators have also tabled a TRIPs-plus ‘access to medicines’ provision, which includes patent term extensions, data exclusivity and patent linkages. This offers companies an ‘access window’ effectively extending the length of patent monopolies while companies seek marketing approval for their drugs in TPP member country markets. The intention of patent linkage is to connect drug regulatory agencies and to require a mandatory check that the marketing of new (e.g. generic) drugs do not infringe on existing patents. Data exclusivity is a period when a patent holder may withhold test data from generic manufacturers thereby constraining the ability to manufacture it (although this may be voluntary for developing countries) [127,128]. Together, these provisions have the potential to restrain access to and increase prices of NCD medicines and technologies in the region.

### Discussion: a research agenda for diagnosing and strengthening regulatory capacity

There are several limitations of this analysis. It reviews the evidence as it applies to a selection of Asian countries, excluding many where risk commodity consumption is also exerting a significant health and economic toll. Nonetheless, we capture countries with the largest population sizes. The market data we have used to describe changes in risk commodity consumption are imperfect measures because these do not capture those commodities manufactured in the home or sold through informal (non-market) productions systems, nor for wastage. However unlike survey data these data are not subject to recall bias, and their abundance allowed for comparisons between countries over time [21]. Most importantly, the paper focuses on risk commodity consumption and does not highlight the ways in which trade liberalization can improve health directly through for example promoting access to fruits and vegetables, or indirectly through contributing to broader economic development. Nor does it elaborate on the indirect effects of trade liberalization on NCDs through, for example, changes in access to health care services or heightened employment insecurity resulting from changing labour market structures [14].

Despite these limitations this article has provided a synthesis of the literature and presented data on the considerable health and economic externalities resulting from risk commodity consumption in the region. It has demonstrated how successive layers of the global trade regime have contributed to the proliferation of the tobacco, alcohol and ultra-processed food industries, and how they might constrain access to NCD medicines across Asia. The findings bring into play pressing questions for future research. How in the context of increasing trade liberalization can public health responses to risk commodity consumption in Asia be strengthened at the global, regional and national levels? We offer some directions for future work on strengthening NCD governance capacity in Asia.

In 2011 the United Nations General Assembly adopted the *Political Declaration on the Prevention and Control of Non-communicable Diseases* (UNPDNCD), the highest multilateral mechanism addressing NCDs [131]. Although trade was referred to as an important sector for intervention in the regional consultations leading up to the UNHLM its inclusion within the Political Declaration was opposed by the US and EU [132]. International standards developed by WHO will, however, be important to protecting domestic policy space to address risk commodities [28,133]. The 2003 *Framework Convention on Tobacco Control* (FCTC) (adopted under Article 19 of the WHO constitution) is a legally binding treaty that may be used to uphold domestic tobacco legislation in trade dispute arbitration, as exemplified recently in arguments by Australia to defend its plain packaging legislation in response to WTO dispute arbitration, although a ruling has yet to be made [134].

For ultra-processed foods and alcohol, however, international agreements are non-binding recommendations (adopted under Article 23), the 2004 *Global Strategy on Diet, Physical Activity and Health*, and 2010 *Global Strategy to Reduce the Harmful Use of Alcohol* respectively. The former states that no provisions in the recommendations should be construed as justification for trade restrictive measures while the latter recognizes the important role of trade as a determinant of alcohol consumption [135,136]*.* The feasibility and approaches for strengthening international standards to address ultra-processed foods and alcohol have been explored elsewhere, and may include the development of more selective mechanisms targeting particular products (e.g. soft drinks) or services (e.g. advertising) as well as standards set by other international organizations including Codex Alimentarius [137–139]. We need to understand much better the role of Asian nations in developing the above and other relevant international standards, particularly explaining any change as their increasing economic and political power leads to greater influence in global health governance more generally [140,141].

Countries may adopt provisions in the aforementioned international agreements as domestic policies so long as they are consistent with international trade obligations – that is they do not arbitrarily or unjustifiably discriminate or act as disguised barriers to trade. Increasing participation in trade negotiations, however, requires countries to strengthen regulatory capacity in terms of monitoring trade agreements and ensuring compliance with international obligations, but also in terms of managing associated risks and ensuring adequate protections for public health [84,142]. Achieving these objectives can be a significant challenge for poorer countries, especially when the negotiating delegations of rich countries are likely to be backed by deep-pocketed TRCC lobbyists and extensive legal teams [143]. They may also struggle to develop the scientific and legal expertise required to evaluate the costs and benefits of entering into trade agreements [144]. This suggests that without the development of such capacities trade agreements have the potential to be an important driver of health inequities between countries in the region [126].

The WHO *Global Action Plan for the Prevention and Control of Noncommunicable Diseases 2013–2020* (GAPNCD) recognizes the role of WHO in offering technical assistance to developing country governments to mitigate the impact of trade agreements on health. Such assistance may be critical to addressing the proliferation of risk commodity industries in Asia, especially in developing countries with limited regulatory capacity. The GAPNCD also calls on the FAO to ‘Support ministries of agriculture in aligning agricultural, trade and health policies’ and on the WTO to ‘…support ministries of trade in coordination with other competent government departments (especially those concerned with public health), to address the interface between trade policies and…noncommunicable diseases’ [145], p74. Although the use of health impacts assessments (HIAs) have been proposed, we do not yet fully understand the efficacy of using HIAs to make trade work for rather than against health in practice [146]. Research is required on the effects of frameworks designed specifically to monitor the impacts of trade agreements on risk commodity consumption, as developed recently in the nutrition space [95]. This also raises research questions around capacity-building for global health diplomacy and the effects of providing developing country officials with the requisite training and skills to participate effectively in trade and health negotiations [147].

The potential opportunities at the regional level are under researched in the current literature. Despite the primacy they give to trade liberalization, ASEAN and the Asia Pacific Economic Cooperation forum have recently demonstrated increased commitment to addressing regional health issues, in particular infectious disease threats [148,149]. As others have noted, however, ASEAN member states have been resistant to attempts to develop regional policy mechanisms that impinge on their national sovereignty. Decision-making processes have been slow due to a ‘rule-by-consensus’ culture and their highly politicized nature (the ‘ASEAN’ way) [149]. The ASEAN Health Ministers Meeting is held every two years yet it has confined its work largely to infectious disease control and disaster preparedness, with agreements to date focused largely on sanitary and phyto-sanitary measures [150]. However, in a joint statement in 2012, ASEAN Plus Three Health Ministers recognized the significant NCD burden in the region and affirmed their commitment to implementing the UNPDNCD [151]. Actions to address NCDs have fallen under the ASEAN Strategic Framework on Health Development (2010-2015) with working groups established to address regional tobacco control and NCDs, but not ultra-processed foods or alcohol [152]. As scholars, we need to understand the extent to which ASEAN and other regional bodies constitute an effective platform for generating regional positions or mechanisms to address trade and risk commodities, especially when one of its key constituent members, Indonesia, is yet to ratify the FCTC.

Asia is also home to ‘light-house’ countries taking unilateral action to address risk commodity consumption. Thailand, for example, has one of the most comprehensive tobacco control regimes globally (although as shown in Figure 1 consumption levels remain relatively high) [108]. It has implemented a hypothecated 2% levy on alcohol and tobacco sales to fund its Thai Health Promotion Foundation [153]. It is also a world leader in addressing health and trade issues. Its National Committee on International Trade and Health, established by the National Health Assembly, brings together officials from ministries of industry, public health, food and agriculture, as well as various professional groups to investigate how trade agreements affect health, to advocate for the inclusion of health in trade negotiations and to coordinate action between concerned agencies [42]. Other governments in the region may consider the establishment of their own ministerial level and intergovernmental bodies with a mandate to address trade and health related issues. As mentioned earlier the WHO and other international organizations have key roles to play in building capacity here. Development assistance for health programmes may also focus work in this area. However, caution is warranted; although the EU has done this recently with assistance for developing South-East Asia nations to establish patent offices, this also strengthens patent compliance in the region and may therefore favour the interests of European pharmaceutical companies (4). Earlier we described how India has adopted a novel domestic patent regulatory regime that offers strong public health protections to improve access to essential NCD medicines. This prompts the need for a seam of research into whether cooperation between countries in order to share such regulatory lessons is or could be important to strengthening policy responses to NCDs across the region [84].

This article has not explicitly demonstrated the mechanisms by which the expanding growth and power of TRCCs facilitate increased risk commodity consumption. The understandings of such mechanisms appears to be theoretically and empirically underdeveloped in the public health literature. The distinct characteristics of corporate activity within the risk commodity industries of Asia also remain opaque. Fundamentally, however, strengthening policy and governance responses to address NCDs will require directly challenging the power of these commercial actors. Globalization enhances the power of TRCCs to influence international and national policy agendas because nation states must increasingly compete with one another to attract and retain the investments and jobs they provide [79,87–89], especially when such companies are among the largest operating in LMICs [20]. This creates a difficult paradox for government regulators who must balance the opportunities for economic development these companies provide and the public health and welfare implications of those investments [20]. Governments of the US, EU and Japan have implemented comprehensive policies and programmes to address risk commodity consumption at home, but TRCCs located in their countries are now driving an NCD pandemic abroad. Trade and investment liberalization may, therefore, act as a mechanism whereby these countries externalize the social and economic costs arising from their risk commodity industries while benefiting from the expatriated profits of the same industries [89].

## Conclusions

In a world characterised by increasing economic globalization it is important to understand how markets and commercial actors operate in ways that are detrimental or beneficial to health [154]. This article has demonstrated how the evolving layers of the international and regional trade regimes have facilitated increased market penetration by transnational tobacco, alcohol and ultra-processed food corporations and thereby driven consumption of these risk commodities in Asia. It has also elaborated on the implications for access to NCD medicines. Increased participation in trade agreements requires governments and public health actors to strengthen regulatory capacity to ensure adequate protections for public health. This article provides a justification for developing such capacity in order to defend public interests against the coordinated and extensive litigation strategies of TRCCs and their representative governments in trade negotiations and disputes [143]. Further research is warranted to understand how this can be achieved at the multilateral, regional and national levels. Without such capacity, trade agreements may become an increasingly important driver of risk commodity consumption, NCDs and health inequities between countries in the Asia region.

## Endnotes

^a^A comprehensive description of the respective risk commodity industries of Asia and the mechanisms by which transnational risk commodity corporations facilitate increased consumption is the subject of a forthcoming article by the same authors. These mechanisms include for example increased market concentration, more intensive competition, more intensive advertising, initial reductions in product pricing and in some cases aggressive lobbying against the adoption of public health control measures.

^b^The constriction of domestic policy space is further facilitated in three ways. First, through the layering of globalized and more difficult processes of making health policy onto existing domestic policy-making processes (procedural constriction). This can result in ‘regulatory chill’ whereby the potential threat of trade sanctions or costly litigation can deter government institutions from initiating policy processes. Second, through limiting the availability of policy instruments available to governments (substantive constriction) including those pertaining to advertising restrictions, mandatory product labelling and product content disclosures. Third, by expediting a shift from public to private provision of goods and services that expands the economic and regulatory power of private relative to public sector actors (structural constriction) [97].

^c^WTO trade agreements most relevant to risk commodities include: (i) General Agreement on Trade in Services (GATS); (ii) Agreement on Agriculture (AoA); (iii) Agreement on the Application of Sanitary and Phytosanitary (SPS) measures; (iv) Agreement on Trade-Related Aspects of Intellectual Property Rights (TRIPS); (v) Technical Barriers to Trade (TBT) Agreement; (vi) Dispute Settlement Understanding; (vii) Agreement on Trade-Related Investment Measures (TRIMS); and, (viii) Agreement on Government Procurement (AGP) [95].
